# Parvovirus B19 DNA Detected in Ovarian Teratomatous Tissue in Anti-NMDAR Encephalitis: A Case Report

**DOI:** 10.3390/v18040405

**Published:** 2026-03-25

**Authors:** Trifon Valkov, Dobroslav Kyurkchiev, Ekaterina Kurteva, Kalina Tumangelova-Yuzeir, Jeliazko Arabadjiev, Vesela Ivanova, Dimitrinka Kisova, Radka Argirova, George Dimitrov, Yordanka Yamakova

**Affiliations:** 1 Department of Infectious Diseases, Medical University of Sofia, Prof. Ivan Kirov Hospital, 1431 Sofia, Bulgaria; 2 Department of Clinical Immunology, Medical University of Sofia, University Hospital “Ivan Rilski”, 1431 Sofia, Bulgaria; dkyurkchiev@medfac.mu-sofia.bg (D.K.); ekurteva@medfac.mu-sofia.bg (E.K.); ktuzeir@medfac.mu-sofia.bg (K.T.-Y.); 3 Acibadem City Clinic, University Hospital “Tokuda”, 1407 Sofia, Bulgaria; 4 Department of General and Clinical Pathology, Medical University of Sofia, 1431 Sofia, Bulgaria; vivanova@medfac.mu-sofia.bg (V.I.); d.kisova@medfac.mu-sofia.bg (D.K.); 5 Department of Clinical Laboratory, Acibadem City Clinic Tokuda Hospital, 1407 Sofia, Bulgaria; radkaargirova@abv.bg; 6 Department of Medical Oncology, Medical University of Sofia, University Hospital “Tsaritsa Yoanna”, 1527 Sofia, Bulgaria; gdimitrov@medfac.mu-sofia.bg; 7 Department Anaesthesiology and Intensive Care, Medical University of Sofia, University Hospital “Aleksandrovska”, 1431 Sofia, Bulgaria; yyamakova@medfac.mu-sofia.bg

**Keywords:** anti-NMDAR encephalitis, autoimmune encephalitis, ovarian teratoma, parvovirus B19, neuroglial tissue, viral persistence, neuroimmunology, paraneoplastic syndromes, young adult, tumor-associated autoimmunity

## Abstract

Background: Anti-N-methyl-D-aspartate receptor (anti-NMDAR) encephalitis is an autoimmune disorder frequently associated with ovarian teratomas in young women. Although infectious triggers have been proposed to contribute to immune activation, direct evidence linking viral presence within tumor tissue to disease pathogenesis remains limited. Case Presentation: An 18-year-old woman presented with acute neuropsychiatric symptoms, fever, gastrointestinal prodrome, and rapidly progressive behavioral disturbance progressing to encephalopathy. Cerebrospinal fluid and blood test results, together with clinical features, supported the diagnosis of anti-NMDAR encephalitis. Imaging identified an ovarian mass, and surgical resection was performed. Histopathology confirmed a mature teratoma containing neuroglial elements. Molecular analysis detected parvovirus B19 DNA within the resected teratomatous tissue. No systemic viremia or active central nervous system viral infection was identified. The patient received immunotherapy combined with tumor removal, with subsequent clinical improvement. Discussion: Ovarian teratomas remain a critical etiologic factor in anti-NMDAR encephalitis and mandate prompt surgical management. Detection of B19 viral DNA within teratomatous neuroglial tissue raises the hypothesis that viral persistence could enhance local immune activation and autoantibody generation. However, in this case polymerase chain reaction positivity does not indicate active infection, and the biological significance of this finding remains uncertain. Conclusions: This case documents rare detection of B19V DNA within an ovarian teratomatous tissue in anti-NMDAR encephalitis. The observation is hypothesis-generating rather than causal; established management priorities remain immunotherapy and tumor resection, and viral nucleic acid detection should be interpreted within the broader clinical context.

## 1. Introduction

Autoimmune encephalitis (AE) is a potentially reversible neuroinflammatory disorder mediated by antibodies targeting neuronal surface or intracellular antigens, resulting in synaptic dysfunction and diffuse brain involvement. It typically presents with acute or subacute onset of altered mental status, behavioral disturbance, cognitive impairment, seizures, and movement abnormalities. Up to 60% of patients report a prodromal illness characterized by fever, malaise, or gastrointestinal symptoms, frequently leading to an initial suspicion of infectious encephalitis [1].

Among autoimmune encephalitides, anti-N-methyl-D-aspartate receptor (anti-NMDAR) encephalitis is the most common subtype, predominantly affecting children and young adults and showing a marked female predominance [2]. The disease often begins with prominent psychiatric manifestations—anxiety, agitation, psychosis, insomnia, or behavioral change—followed by seizures, dyskinesias, speech dysfunction, impaired consciousness, and autonomic instability. Because early symptoms may mimic primary psychiatric disorders, diagnosis is frequently delayed [3].

A well-recognized paraneoplastic association exists between anti-NMDAR encephalitis and ovarian teratomas. Tumors are identified in approximately 27–58% of affected women over 18 years of age, with frequency increasing after puberty. These teratomas contain neuroglial tissue expressing NMDA receptors and lymphoid aggregates capable of intratumoral antibody production, thereby triggering the autoimmune response [4]. Notably, associated teratomas are often small, predominantly solid, and may lack classic radiologic features, necessitating careful imaging and pathological evaluation. Tumor removal combined with immunotherapy substantially improves neurological outcomes, accelerates recovery, and reduces relapse risk, underscoring the importance of systematic tumor screening in female patients [5].

Despite this association, a substantial proportion of cases are non-paraneoplastic, and rare triggers have been described. Parainfectious immune activation following viral illness has been proposed as a precipitating mechanism. Parvovirus B19 (B19V), although uncommon, has been reported both as a potential trigger of anti-NMDAR encephalitis and as an independent cause of viral encephalitis with overlapping neuropsychiatric features [6]. Detection of viral DNA in cerebrospinal fluid therefore does not exclude autoimmune encephalitis, and parallel evaluation for neuronal antibodies remains essential when clinical features suggest an immune-mediated process [7].

AE accounts for approximately 4% of acute encephalitis cases, and in young individuals, anti-NMDAR encephalitis may exceed the frequency of any single viral cause [8]. Early recognition is critical, as prompt immunotherapy—including corticosteroids, intravenous immunoglobulin, or plasmapheresis, followed by second-line agents such as rituximab when needed—significantly improves outcomes [9]. Here, we present the case of a previously healthy young woman with rapid neuropsychiatric deterioration following a prodromal febrile illness, highlighting diagnostic challenges and emphasizing rare etiologic considerations in the evaluation of suspected autoimmune encephalitis.

## 2. Case Presentation

An 18-year-old previously healthy woman was admitted on 30 May 2025 with a working diagnosis of other encephalitis, myelitis, and encephalomyelitis (ICD-10 G04.8). She had no known comorbidities. Because of her altered mental status, the clinical history was obtained from her mother. The illness began acutely on 25 May 2025. During the night of 25–26 May, the patient experienced severe malaise and awoke drenched in sweat. On 26 May, she developed diarrhea without blood or mucus, subjective fever, nausea, and generalized weakness. Despite ongoing symptoms, she attended her high school prom dinner later that day, where she reported consuming a small amount of wine, taking several puffs from an electronic cigarette, and not eating. She denied illicit drug use. That evening, her temperature reached 38 °C, and gastrointestinal symptoms persisted with nausea and multiple episodes of vomiting. On 27 May, she became markedly confused, agitated, and behaviorally inappropriate. On 28 May, due to worsening symptoms, she was taken to a private medical facility where intravenous glucose–saline infusions were administered; documentation from this visit was unavailable. On 29 May, persistent agitation, severe anxiety, several days of insomnia, emotional lability with unprovoked crying, and ongoing fever prompted presentation to the Emergency Department at Alexandrovska University Hospital. Psychiatric evaluation documented hyperthymia, grandiose ideation, and paranoid thoughts of being monitored. She received antihistamine therapy and haloperidol for behavioral control prior to neurological assessment. A neurological consultation was obtained, and she was admitted to the Department of Neurology. Lumbar puncture revealed cerebrospinal fluid pleocytosis with mild protein and glucose abnormalities (Table 1). Urine toxicology screening was negative. On 30 May 2025, she was referred for hospitalization at a specialized infectious diseases hospital for further evaluation and management.

At admission, her general condition was severely impaired. She was conscious but disoriented to time and place, psychomotor-agitated, and subfebrile (37.5 °C). Respiratory examination revealed vesicular breath sounds with a respiratory rate of 29/min. Cardiovascular examination showed blood pressure 138/104 mmHg and heart rate 128 beats/min with clear heart sounds. Neurological examination demonstrated no signs of meningeal irritation. Pupils were equal and reactive to light; there was no nystagmus, diplopia, or facial asymmetry. The tongue was midline. A left extensor plantar response (Babinski sign) was present. Palmomental reflexes were bilaterally positive, as were Hoffmann and Trömner signs. Deep tendon reflexes were diffusely brisk with expanded reflexogenic zones and polykinetic responses in the lower extremities up to clonus, accompanied by spastic hypertonia in the lower limbs. Speech was absent, and cognition was markedly slowed with delayed command execution.

On 30 May 2025, she experienced two generalized tonic seizures: the first at approximately 16:30, lasting 90 s, and the second at 21:00, lasting 40 s. Continuous anticonvulsant therapy with valproate was initiated. A repeat lumbar puncture on 2 June included microbiological studies: throat culture grew Haemophilus parainfluenzae (10^4^ CFU), while CSF PCR testing was negative for Streptococcus agalactiae, Cryptococcus neoformans/gattii, Listeria monocytogenes, Neisseria meningitidis, Mycobacterium tuberculosis, HHV-6, HSV-1, adenovirus, B19V, Mycoplasma pneumoniae, enteroviruses, mumps virus, and human parechovirus.

The patient received comprehensive therapy including rehydration, osmotherapy, antibacterial and antiviral agents, corticosteroids, anticonvulsants, and symptomatic treatment. Parallel serum and cerebrospinal fluid (CSF) testing for autoimmune encephalitis antibodies was performed using an indirect immunofluorescence assay (“Autoimmune Encephalitis Mosaic 6,” Euroimmun, Lübeck, Germany) according to the manufacturer’s instructions.

**Figure 1:**
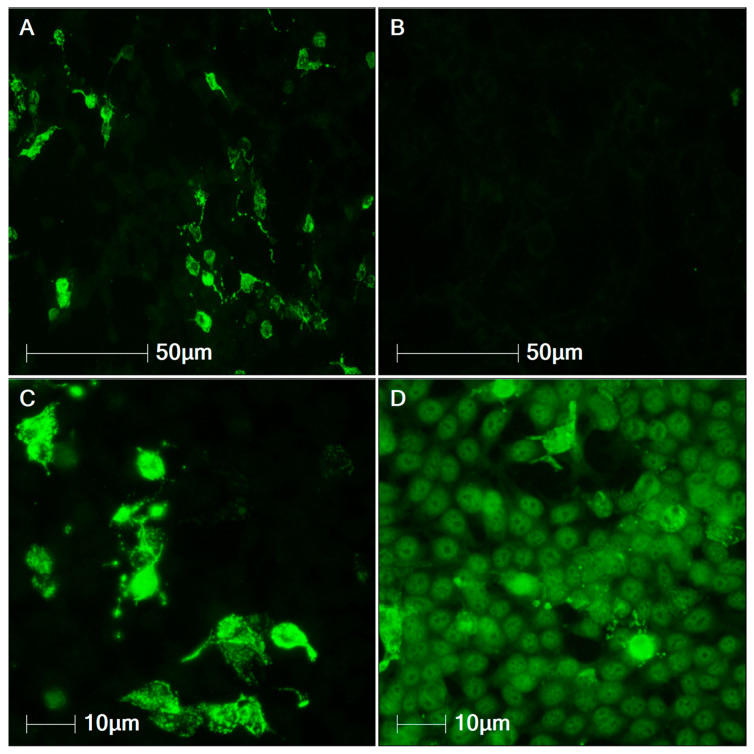
Immunofluorescence with anti-NMDAR antibodies. Detection of anti-NMDAR antibodies by indirect immunofluorescence. The photomicrograph images represent: (**A**) Positive control (Original magnification, 200×). (**B**) Negative control (Original magnification, 200×). (**C**) CSF (Original magnification, 400×). (**D**) Serum (Original magnification, 400×).

Indirect immunofluorescence performed on 2 June 2025 detected anti-NMDAR autoantibodies in both CSF (titer 1:32) and serum (titer 1:100) (Figure 1).

**Figure 2:**
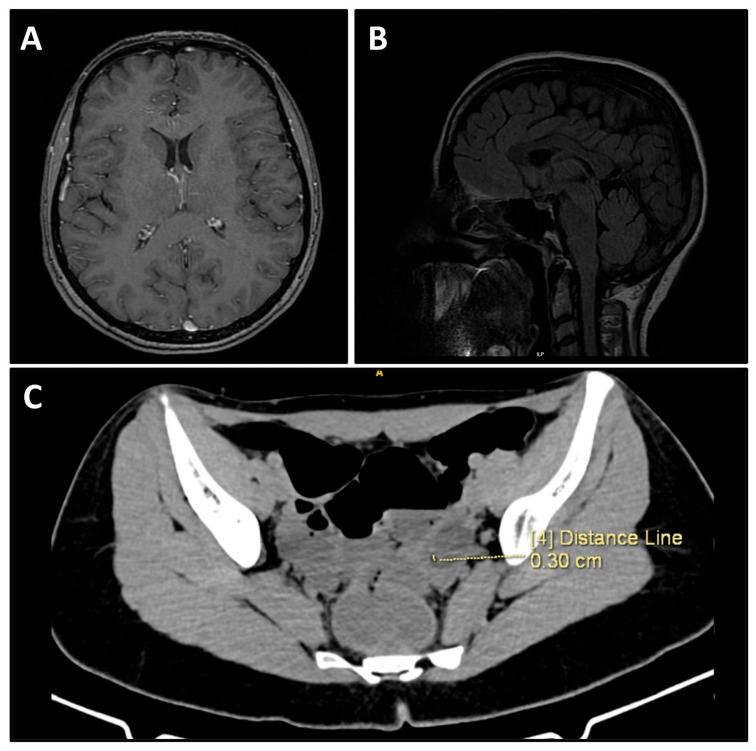
Imaging diagnostics. (**A**) Normal axial MRI of the brain in T1. (**B**) Normal sagittal MRI of the brain in T2 FLAIR. (**C**) CT scan of the lesser pelvis demonstrating a 27 × 19 mm cystic lesion in the left ovary.

Additional immunofluorescence analysis on 3 June 2025 demonstrated findings consistent with inflammatory meningeal changes. Brain MRI showed no structural abnormalities (Figure 2A,B). Neurology consultation recommended high-dose intravenous methylprednisolone (1000 mg/day for 3 days followed by 500 mg/day). Due to clinical severity, she was transferred on 5 June 2025 to the intensive care unit of Alexandrovska University Hospital for continued management. Contrast-enhanced CT of the abdomen and pelvis performed on 5 June 2025 revealed a 27 × 19 mm cystic lesion in the left ovary containing fat and calcifications, most consistent with a mature cystic teratoma (Figure 2C).

**Figure 3:**
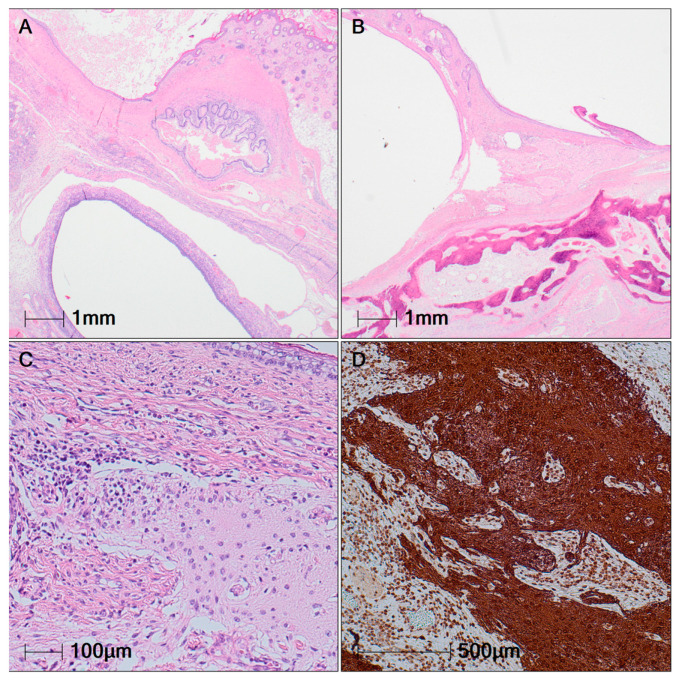
Histological findings in the left ovary. (**A**) Teratoma, H&E original magnification, 20×. (**B**) Teratoma—mature bone formation, H&E original magnification, 20×. (**C**) Neuroglial tissue, H&E original magnification, 200×. (**D**) GFAP immunostaining, original magnification, 100×.

On 9 June 2025, the patient underwent surgical treatment consisting of left adnexectomy with tumor extirpation. Histopathological examination confirmed the diagnosis of a mature cystic teratoma, composed of well-differentiated tissues derived from all three germ layers. The predominant component was squamous epithelium with skin adnexal structures, accompanied by mesenchymal elements including adipose tissue, cartilage, and bone formation (Figure 3A,B). A key finding supporting the clinical manifestation of anti-NMDAR encephalitis was the presence of neuroglial tissue surrounded by lymphoid aggregates. Immunohistochemical staining for glial fibrillary acidic protein (GFAP) was performed, highlighting areas of glial differentiation (Figure 3C,D).

**Figure 4:**
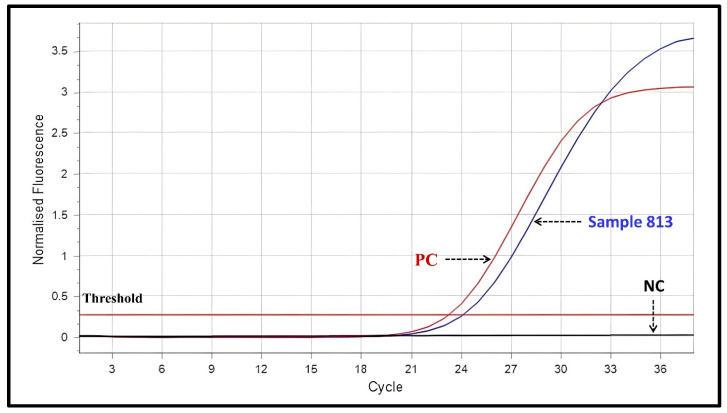
Multiplex real-time PCR analysis performed using the Bosphore CNS Mini Panel Kit v2 (Anatolia Geneworks, Turkey; CE-marked) for detection of viral DNA/RNA in tissue/biopsy and CSF samples. Fluorescence detection was carried out using FAM, HEX, Texas Red, and Cy5 channels. In PCR Master Mix 2, amplification targets included Human Herpesvirus 6 (HHV-6; U57 gene, FAM channel), B19V (NS gene, HEX channel), and Human Herpesvirus 7 (HHV-7; glycoprotein H gene, Cy5 channel). The HEX channel demonstrated a positive signal for B19V in sample 813. Although the HEX channel may also detect VZV (mix 1) and Parechovirus (mix 3), these mixes were not used in the present experiment. The diagnostic cut-off for positivity was defined as Ct ≤ 35. Abbreviations: PC, positive control; NC, negative control.

On 19 June 2025, qualitative PCR testing performed on deparaffinized teratoma tissue lysate evaluated for VZV, B19V and parechovirus with HEX signal being positive for B19V DNA (Figure 4). By 3 July 2025, the patient was discharged in good general condition—conscious, fully oriented, and without residual neurological deficits or qualitative or quantitative disturbances of consciousness. A follow-up brain MRI performed on 20 August 2025 was normal. Repeat lumbar puncture with indirect immunofluorescence on 28 July 2025 demonstrated persistent anti-NMDAR antibodies in both serum (titer 1:32) and CSF (titer 1:10).

## 3. Discussion

This case highlights an exceptionally rare finding: the detection of B19V DNA within resected ovarian teratoma tissue in a patient with confirmed anti-NMDAR encephalitis. To date, there is no established evidence that B19V infection of ovarian teratomas predisposes to or triggers anti-NMDAR encephalitis. The two well-documented triggers of anti-NMDAR encephalitis are ovarian teratomas containing neuroglial tissue and herpes simplex virus encephalitis (HSVE); B19V has not been recognized among validated infectious precipitants [4,10].

The pathogenesis of teratoma-associated anti-NMDAR encephalitis is well characterized. Neuroglial elements within the tumor express NMDA receptors, particularly the GluN1 subunit, which initiate autoantibody production [11]. Histopathologic studies demonstrate colocalization of neuroglial tissue and tertiary lymphoid structures with active germinal centers in the majority of NMDAR-associated teratomas, in contrast to control teratomas [12]. Intratumoral B cells are capable of synthesizing anti-NMDAR antibodies in vitro, supporting the concept of the tumor as an active immunologic niche. These antibodies can cross-react with physiological NMDA receptors expressed on central nervous system neurons. Antibody binding induces receptor internalization through endocytosis, resulting in functional receptor loss. Surgical resection removes the antigenic stimulus and is associated with accelerated clinical recovery and a reduced risk of relapse, as observed in our patient.

In contrast, viral-triggered anti-NMDAR encephalitis is most convincingly documented following HSVE. Approximately 27% of prospectively followed HSV encephalitis patients develop autoimmune encephalitis within 2–16 weeks, with NMDAR antibodies detected in nearly two-thirds of cases [4]. Beyond HSV, other viral associations—including Epstein–Barr virus (EBV), Japanese encephalitis virus, tick-borne encephalitis, and human herpesvirus-7—have been reported primarily in case reports or small series, and causality remains less firmly established [13,14]. Importantly, none of these reports have demonstrated viral infection of teratoma tissue itself as a mechanistic contributor to antibody generation.

B19V has been implicated in autoimmune phenomena through proposed mechanisms such as molecular mimicry, cross reactivity, apoptosis-induced autoantigen exposure, and immune activation mediated by the VP1 unique region phospholipase activity [7]. Neurologically, B19V can cause direct viral encephalitis and has been associated in rare instances with autoimmune encephalitides, including a reported case of GABA receptor encephalitis [15,16]. However, no published data have demonstrated B19V infection within ovarian teratomas or linked such infection to teratoma-mediated anti-NMDAR encephalitis.

The isolation of B19V DNA from the teratoma tissue in our patient therefore represents an unusual and, to our knowledge, previously undocumented observation. Several theoretical mechanisms could be considered. If B19V were to infect neuroglial components within the teratoma, viral replication or persistence might promote local inflammation, neuronal degeneration, and enhanced antigen release, potentially amplifying NMDAR-directed immune responses. Alternatively, viral-induced immune activation could facilitate germinal center maturation within the tumor microenvironment, promoting affinity maturation and sustained autoantibody production. Molecular mimicry between viral proteins and neuronal antigens is another hypothetical pathway. Nevertheless, these possibilities remain speculative, as current evidence does not support B19V as a recognized trigger of anti-NMDAR encephalitis, nor does it establish that viral presence within teratoma tissue is pathogenic rather than incidental.

An alternative interpretation is that detection of B19V DNA reflects a prior systemic infection with subsequent viral persistence in tumor tissue, without direct causal relevance. Parvovirus B19 is known to exhibit prolonged DNA persistence in multiple tissues following acute infection, even in immunocompetent individuals [17]. Therefore, the presence of viral genetic material does not necessarily indicate active infection or mechanistic involvement in autoimmunity. In this case, real-time PCR targeted the B19V NS gene, a multifunctional viral protein regarded as a “double-edged sword,” as it is essential for efficient viral replication while also contributing to the pathogenesis of B19V-associated inflammatory and autoimmune disorders [18].

Clinically, this case underscores several key principles. The presence of an ovarian teratoma remains a central etiologic factor in young women with anti-NMDAR encephalitis and warrants prompt surgical resection irrespective of concomitant infectious findings. Detection of viral nucleic acids—whether in cerebrospinal fluid or, as in this case, within tumor tissue—must be interpreted cautiously and in the context of the overall clinical picture. Standard management priorities remain timely immunotherapy and tumor removal, while antiviral treatment should be reserved for cases with evidence of active systemic or central nervous system infection.

This report is limited by its single-case design, precluding conclusions regarding causality or prevalence, and by the absence of control teratoma specimens to determine whether B19V DNA detection exceeds expected background tissue persistence. PCR positivity alone does not establish active infection, as no viral transcriptional activity, protein expression, or cytopathic effect was demonstrated, despite spatial localization of viral material within neuroglial tumor tissue. No mechanistic studies were performed to assess whether viral presence influenced local immune activation, and contamination or passive leukocyte carriage cannot be fully excluded. Additionally, the temporal relationship between prior parvovirus exposure and encephalitis onset remains undefined. Accordingly, this finding should be interpreted as hypothesis-generating rather than evidence of a causal pathogenic role. Nonetheless, the case documents a rare instance of B19V DNA identified within an ovarian teratoma in a patient with anti-NMDAR encephalitis.

## 4. Conclusions

This case documents an unusual detection of B19V DNA within ovarian teratoma tissue in a patient with anti-NMDAR encephalitis. While biologically intriguing, current evidence does not support a causal relationship between B19V infection and teratoma-associated anti-NMDAR encephalitis. The finding should be regarded as hypothesis-generating. Systematic evaluation of viral presence in teratomas, coupled with spatial localization and functional immunologic studies, would be required to clarify whether viral infection of tumor tissue can modulate autoimmune risk.

## Figures and Tables

**Table 1:** CSF analyses.

Date	Protein (g/L)	Cells/L	Glucose (mmol/L)
29 May 2025	0.36	70 × 10^6^	2.75
30 May 2025	0.28	17 × 10^6^	3.16
28 July 2025	0.21	6 × 10^6^	2.85

## Data Availability

The dataset presented in this article was obtained during routine clinical patient care and is not readily available because of patient privacy protection.

## References

[1] de Bruijn M., Leypoldt F., Dalmau J., Lee S.T., Honnorat J., Clardy S.L., Irani S.R., Easton A., Kunchok A., Titulaer M.J. (2025). Autoimmune encephalitis. Nat. Rev. Dis. Primers.

[2] Dalmau J., Graus F. (2018). Antibody-Mediated Encephalitis. N. Engl. J. Med..

[3] Yucel Y., Sidow N.O., Yilmaz A. (2025). Approach and overview of autoimmune encephalitis: A review. Medicine.

[4] Dalmau J., Armangué T., Planagumà J., Radosevic M., Mannara F., Leypoldt F., Geis C., Lancaster E., Titulaer M.J., Rosenfeld M.R. (2019). An update on anti-NMDA receptor encephalitis for neurologists and psychiatrists: Mechanisms and models. Lancet Neurol..

[5] Nha P.B., Tu N.P., Ha N.V., Hien D.T.T., Phuong N.T.T., Son N.A., Hoang N.T. (2025). Anti-NMDA receptor autoimmune encephalitis associated with ovarian teratoma: A case series and literature review. Int. J. Gynaecol. Obstet..

[6] Kerr J.R. (2016). The role of parvovirus B19 in the pathogenesis of autoimmunity and autoimmune disease. J. Clin. Pathol..

[7] Tzang C.C., Chi L.Y., Lee C.Y., Chang Z.Y., Luo C.A., Chen Y.H., Lin T.A., Yu L.C., Chen Y.R., Tzang B.S. (2025). Clinical implications of human Parvovirus B19 infection on autoimmunity and autoimmune diseases. Int. Immunopharmacol..

[8] Sharma K., Khandia R., Shrivastava R., Nema R.K., Mishra S., Kanwar R.K., Raut A.A., Agrawal A., Gupta V., Pandey M.K. (2025). Exploring the link between parvovirus B19 and encephalitis: A systematic review and comprehensive meta-analysis of molecular and serological evidence. Virol. J..

[9] Titulaer M.J., McCracken L., Gabilondo I., Armangué T., Glaser C., Iizuka T., Honig L.S., Benseler S.M., Kawachi I., Martinez-Hernandez E. (2013). Treatment and prognostic factors for long-term outcome in patients with anti-NMDA receptor encephalitis: An observational cohort study. Lancet Neurol..

[10] Swayne A., Warren N., Prain K., Gillis D., Wong R., Blum S. (2022). Analysing triggers for anti-NMDA-receptor encephalitis including herpes simplex virus encephalitis and ovarian teratoma: Results from the Queensland Autoimmune Encephalitis cohort. Intern. Med. J..

[11] Vilaseca A., Dalmau J.O., Benarroch E. (2025). What Are the Proposed Mechanisms Contributing to the Manifestations of Anti-NMDAR Encephalitis?. Neurology.

[12] Nolan A., Buza N., Margeta M., Rabban J.T. (2019). Ovarian Teratomas in Women With Anti-N-methyl-D-Aspartate Receptor Encephalitis: Topography and Composition of Immune Cell and Neuroglial Populations Is Compatible With an Autoimmune Mechanism of Disease. Am. J. Surg. Pathol..

[13] Zhan X., Zhang X., Chen X., Niu X., Xuan T., He J., Ren Y., Meng Y., Guo T., Li H. (2025). Anti-NMDAR encephalitis triggered by EBV and HSV-1: A case report and literature review. Medicine.

[14] Yang J., Wu P., Liu X., Xia H., Lai Z. (2021). Autoimmune Encephalitis With Multiple Auto-Antibodies With Concomitant Human Herpesvirus-7 and Ovarian Teratoma: A Case Report. Front. Med..

[15] Schwenkenbecher P., Skripuletz T., Lange P., Dürr M., Konen F.F., Möhn N., Ringelstein M., Menge T., Friese M.A., Melzer N. (2021). Intrathecal Antibody Production Against Epstein-Barr, Herpes Simplex, and Other Neurotropic Viruses in Autoimmune Encephalitis. Neurol. Neuroimmunol. Neuroinflamm..

[16] Duc N.D., Hong Anh L.N., Hong Khanh L.N., Bach N.D. (2026). Parvovirus B19 encephalitis in adults: A case report from Vietnam. Virol. J..

[17] Servant-Delmas A., Morinet F. (2016). Update of the human parvovirus B19 biology. Transfus. Clin. Biol..

[18] Jalali S., Farhadi A., Rafiei Dehbidi G., Farjadian S., Sharifzadeh S., Ranjbaran R., Seyyedi N., Namdari S., Behzad-Behbahani A. (2022). The Pathogenic Aspects of Human Parvovirus B19 NS1 Protein in Chronic and Inflammatory Diseases. Interdiscip. Perspect. Infect. Dis..

